# Exploring Compassionate Care in Virtual Rehabilitation: Qualitative Study

**DOI:** 10.2196/59157

**Published:** 2025-05-22

**Authors:** Parminder Flora, Angela Tobia, Lee Verweel, Bernice Lau, Janet Campbell, Arezoo Eshraghi, Steven Dilkas, Roger Goldstein, Patricia Raulino, Crystal MacKay

**Affiliations:** 1 West Park Healthcare Centre, University Health Network, Toronto, ON, Canada; 2 KITE Research Institute, University Health Network, Toronto, ON, Canada; 3 School of Rehabilitation Therapy, Queen's University, Louise D Acton Building, 31 George Street, Kingston, ON, K7L 3N6, Canada, 1 613-533-6103

**Keywords:** compassion, rehabilitation, virtual care, virtual rehabilitation, limb loss, chronic obstructive pulmonary disease, qualitative interviews

## Abstract

**Background:**

Virtually delivered health care services can offer numerous benefits, and the demand for virtual care continues to grow among subgroups facing mobility challenges. The experience of compassion in health care is linked to patient satisfaction and clinical outcomes; however, this link in virtual rehabilitation settings is underexplored.

**Objective:**

The objectives of this study were to explore what compassionate care means to rehabilitation patients in a virtual rehabilitation context and explore patients’ experiences of how the technology associated with virtual rehabilitation impacted their experience of care.

**Methods:**

We conducted one-on-one semistructured qualitative interviews with patients with limb loss and chronic obstructive pulmonary disease. A reflexive thematic analysis approach was used to generate domain summaries and initial themes across the sample. Themes were generated following analytic work over a series of discussions within the research team.

**Results:**

Sixteen interviews were conducted. Four themes illustrating participants’ perceptions of compassionate care were generated: (1) features of compassionate care include feeling valued, connected, and cared for by the health care provider; (2) threats to compassionate care in virtual rehabilitation; (3) facilitating compassion in virtual rehabilitation through preparation; and (4) benefits of virtual care.

**Conclusions:**

Patient perceptions of compassionate care in a virtual rehabilitation setting may be impacted by the behaviors and communication of providers. Provider training and preparation and the personal connections formed with their patients may impact compassionate care experiences.

## Introduction

Virtual care refers to health care services that are delivered remotely through the use of communication and digital technologies in order to benefit patients and maximize the quality of patient care. The COVID-19 pandemic resulted in an increased use of virtual care in Canada, with broad adoption across provider groups and types of chronic disease [1]. The increased demand for virtual care during the COVID-19 pandemic is expected to continue post pandemic [2], especially for individuals and subgroups that experience challenges attending in-person, for example, due to mobility challenges [3,4].

A growing body of literature about virtual care has described its numerous benefits, including convenient access to quality care, reduced time and cost of traveling to appointments, and improved access for rural or isolated communities [5]. Virtual care is also particularly beneficial for patient populations with a high disease burden [5]. Some benefits for providers include improved care efficiency (eg, seeing more patients and fewer missed appointments) and more frequent contact between patients and providers [5,6].

There are also limitations to virtual care, such as difficulty establishing a therapeutic connection between providers and patients [7], the absence of physical examination [5,8], limited availability of physical equipment, absent or inadequate cellular services, and the costs associated with training personnel to implement virtual care [5,6,8].

Compassion is an important cornerstone of quality health care [9-11] and a critical element of person-centered care. Compassion has been defined in several ways, including recognizing suffering in others, understanding the humanity of this suffering, feeling emotionally connected with the suffering person, tolerating difficult feelings that arise, and acting or being motivated to act to help the person [12]. Compassion has been associated with improved patient satisfaction, compliance, and clinical outcomes [13-16]. According to Hodges et al [17], digital tools can shape individuals’ expectations, actions, and behaviors; impact the patient-provider relationship; and shape the experience of compassion in health care. As we continue to use virtual care options, it is important to ensure that the care offered virtually is as compassionate and of high quality as the care offered in person.

In the rehabilitation setting, compassion is important for person-centered, empathetic care [18]. Although the importance of compassion in a rehabilitation setting is well recognized, there is very limited information on how compassion is communicated when the process is virtual. Individuals who experienced lower limb amputation (LLA) and individuals with chronic obstructive pulmonary disease (COPD) are examples of populations with chronic conditions who benefit from rehabilitation services to improve symptoms, function, mobility, and health-related quality of life. Exploring the experiences of individuals with LLA and COPD receiving virtual rehabilitation will provide valuable insights to inform the delivery of compassionate, person-centered care in practice. The 2-fold objectives of this study are (1) to explore what compassionate care means to patients living with limb loss and COPD who receive virtual rehabilitation consultations and (2) to explore patients’ experiences of how the technology associated with virtual rehabilitation impacted their experience of care.

## Method

### Study Design

We adopted an interpretive descriptive approach [19]. Interpretive description is the investigation of a clinical phenomenon of interest for the purpose of capturing themes and patterns within subjective perceptions and generating an interpretive description to inform clinical understanding. We used the COREQ (Consolidated Criteria for Reporting Qualitative Research) guidelines.

### Ethical Considerations

This study received research ethics approval from the Joint Research Ethics Board at West Park Healthcare Centre (REB number: 22‐005-WP). Informed consent was obtained from all participants prior to study participation and all data was de-identified. Participants received an honorarium of CAD $25 (US $19.59) for completing the interview. The conversion rate during the study period ranged between US $0.7203 and $0.7835.

### Recruitment

Participants were recruited from a rehabilitation hospital in Ontario, Canada. Individuals who were over age 18 years and had participated in at least one virtual care appointment at the rehabilitation center were eligible to take part in the study. Participants were required to be able to communicate in English and tolerate participating in a 45- to 60-minute interview.

For feasibility reasons, a convenience sampling strategy was used. Participants with limb loss or COPD were recruited, as these clinical populations represented the majority of individuals who were offered virtual care in the rehabilitation setting. Sampling from 2 subgroups was appropriate for this study because the research focus was on the experience of compassion during the virtual care interaction, rather than on any condition-specific issues. Where experiences or perceptions vary between subgroups, this is described in the Results section.

Individuals who consented to be contacted for the study and had completed a virtual visit were approached to participate in the study. Informed consent was collected prior to an interview taking place.

### Data Collection

One-on-one interviews using a semistructured interview guide were conducted remotely via telephone or Zoom. Interviews were conducted by one researcher (BL). The interview guide was developed by the research team based on previous literature (see Textbox 1).

Textbox 1. **Interview guide.**

1. Can you first briefly tell me about yourself and what led to your need for rehabilitation?
2. I understand you had a virtual or remote consultation with a health care provider related to your condition or disability. Tell me about that experience.
3. Tell me about what it was like having your consultation with the team virtually.
4. What worked well about the virtual consultation?
5. What did not work well about the virtual consultation?
6. We often talk about the importance of compassion in health care. What do you understand about the word compassion when you hear it?

Interview questions were open-ended, exploring virtual care experiences, including what worked well and what did not work well, the meaning of compassion, and the experience of compassionate care. Probes were used to elicit more in-depth information and clarify participant responses. After each interview, the interviewer wrote supplementary field notes to allow emerging insights to be incorporated in the ongoing data collection. Data collection and analysis were iterative. Demographic and clinical information was collected to describe the participants. Interviews ranged between 25 and 48 minutes.

Data were collected until researchers were satisfied that data were comprehensive enough with sufficient depth of data to identify recurrent thematic patterns in addition to having breadth of experiences in the context of the study setting. Interviews were recorded and transcribed verbatim. NVivo (Lumivero) qualitative software program was used to organize the data.

### Analysis

A reflexive thematic analysis approach was used to generate domain summaries and initial themes, or patterns of shared meaning united by a core concept [20]. A coding framework was developed following data familiarization [20]. Two researchers familiarized themselves with the data by reading transcripts (BL and CM) before meeting to develop a preliminary codebook with codes and code descriptions. The codebook was used to code 2 additional transcripts, and revisions were made to the codebook. Subsequently, 2 researchers (BL and PF) coded each transcript. Incorporating 2 researchers allowed us to explore multiple assumptions or interpretations of the data. Memos were written as part of the analytic process to encourage reflexivity. Field notes were written after every interview, and notes were summarized and discussed.

Two reviewers (PF and AT) reviewed all codes and separately generated domain summaries before meeting approximately 5 times to discuss and record their interpretations of the data. Themes were generated inductively during this process. Following the generation of initial themes and domain summaries, 3 team meetings between researchers (PF, AT, and CM) were held to question one another, discuss and refine themes, clarify language, and interrogate the data to consider alternative interpretations.

Consistent with reflexive thematic analysis, the researchers played an active role in knowledge production and generation of themes [20] and considered the interpretation of the data. Themes were generated following analytic work over a series of discussions and were actively created through creative labor involving coding, discussion, questioning, and decision-making [20]. Memos and diagrams were generated to summarize and interpret the data. Demographic and clinical information was collected and summarized using descriptive statistics.

## Results

### Participants

Sixteen participants consented to take part in the study and completed the interview. The sample consisted of individuals with limb loss (n=7) and individuals with COPD (n=9). A description of the study sample is provided in Table 1.

**Table 1.** Demographic data.

Characteristic	LLA^a^ (n=7), n	COPD^b^ (n=9), n	Total (n=16), N (%)
Gender			
Female	1	4	5 (31)
Male	5	4	9 (56)
Employment status			
Full time	0	1	1 (6)
Retired	5	5	10 (63)
Unable to work	1	2	3 (19)
Education (highest)			
High school completed	1	3	4 (25)
Some college or university	3	2	5 (31)
College or university completed	2	3	5 (31)
Living arrangement			
Alone	0	2	2 (12.5)
Spouse or partner	3	3	6 (37.5)
Children	1	1	2 (12.5)
Spouse and children	1	1	2 (12.5)
Parents	1	0	1 (6)
Sibling	0	1	1 (6)
Age (years), mean (SD)	61.33 (15.77)	70 (10.54)	66.29 (13.24)

a LLA: lower limb amputation.

b COPD: chronic obstructive pulmonary disease.

c Responses to demographic questions were provided by 14 of 16 study participants. Two participants could not be reached following the interview to complete the demographic questionnaire; therefore, data for those participants are missing.

Four themes were generated to illustrate participants’ perceptions of compassionate care based on their experiences with virtual care in a rehabilitation setting.

### Theme 1: Features of Compassionate Care: Feeling Valued, Connected, and Cared for by the Health Care Provider

#### Overview

This theme characterizes participants’ perceptions of the key features of compassionate care. While the features may not be unique to virtual care, they were seen as important components of compassion in the context of virtual rehabilitation. Several participants described compassion as a feeling of being taken care of, being heard, and being validated. Compassion was equated to caring, kindness, understanding, and warmth. They described compassion as positive, personal, and individualized, not judgmental or offensive. One participant indicated compassion made them feel valuable, like their life is worth something. Reflecting on their experience of compassionate care, one participant stated:

I feel good. I mean it’s very, you know, it’s you feel safe. You feel looked after. You feel heard. It’s very helpful and I think it can - overall contributes to the healing process if you’ve got someone who cares.

[Participant 2]

The following subthemes further describe the core components of compassionate care from the participants’ perspectives.

#### The Caring Health Care Provider

A health care provider’s (HCP’s) ability to offer empathy, reassurance, caring, and attentiveness influenced participants’ experience of compassion. Being caring was most often associated with compassion. For example, one participant stated having a caring and empathetic HCP was more important than other aspects of bedside manner. Caring was described as the effort that HCPs made to show patients they are valued. This included caring actions such as remembering specific details about the patient and asking questions. When participants reflected on instances of genuine caring, their accounts highlighted the importance of authenticity in caring and the importance of an HCP being curious, observant, and attentive and having genuine concern about how their patients are doing. One participant shared this perspective about caring for HCPs and compassion:

To me compassion is authentically, authentic concern and care for an individual. You can put it on and pretend it, you can fake it, you could, but compassion is when it’s authentic genuine care and concern for the wellbeing of someone else and you want the very best for them and the very best outcome for them.

[Participant 3]

Reassurance went hand in hand with caring and was also an important part of compassion. Many participants indicated they felt reassured when HCPs addressed their questions or concerns and went out of their way to be caring. Reassuring words and actions from the HCP helped establish comfort and trust, and made the patient feel like they could turn to and trust the HCP. Many participants shared stories about a difficult moment when an HCP was reassuring to them—participants valued this and interpreted that as compassion. One patient described that compassion involves the HCP understanding what is going on with the patient and advising and acting from that understanding.

#### Patient-Centered Behaviors: HCPs’ Willingness to Help

Participants sensed compassion when HCPs seemed motivated to take action to care for them and were willing to help. Compassion was expressed as actions or words and involves “doing the little things” and “going the extra mile.” Participants shared:

So, it can be actions. It can be words. It can be just making, you know, those, a lot of time it’s words, just making sure like you know, if there’s anything you need at all you just, please, please don’t hesitate to call and let us know. We’re here for you. That kind of extra bit.

[Participant 3]

I’m not very tech savvy, so yeah, the pictures I sent but I didn’t send it to the right spot. So, then actually the doctor was on the phone with me and said, okay, this is how you do it and he started to help me, and we got it done…Yeah, so he was very, very…he’s very helpful in all aspects…And anything that you need or anything – any concerns that you have, he addresses them…I mean usually they don’t – doctors don’t give you that kind of care, like, you know what, they brush you off and say okay, well you know what, you’re gonna have to figure out – maybe get somebody to help you, no, he helped me…But yeah, step by step. Above and beyond.

[Participant 6]

And then with the virtual class now, one lady she does exercises with you and then there’s another lady. I think she’s just watching everyone to make sure everybody’s okay. And if she sees that you look kind of distressed, she’ll call you up and say…She’ll say like I think you should just stop a minute, …they show compassion to everyone to make sure you’re okay…You know, it’s not just, okay, do this, do that and that, okay, next do this, do that. No. They make sure you’re okay.

[Participant 14]

Willingness to help involved a genuine interest from an HCP for doing things to improve patients’ well-being, including taking a step beyond traditional duties and building personal relationships. Many participants provided examples of HCPs exhibiting a willingness to help, which made participants feel cared for and motivated to get better. Participants consistently expressed that compassion felt like it was more than just doing a job and should not feel like it is a business. One participant stated:

To me, I never – I never really felt that, oh, you know, this is just – you’re just a number and here we go. I find that they’re very compassionate and they’re very helpful to what you need. And I never felt brushed off, like, never rushed.

[Participant 6]

#### HCP Communication

Effective communication by the HCP helped participants feel compassion during virtual care appointments. Participants valued a HCP with good communication skills (eg, could “carry a conversation well”), who was personable, social, and attentive. Participants also felt better cared for when the HCP was organized, spoke clearly, and could be easily understood, and when the HCP made an effort to have a personal connection and to make patients feel comfortable (eg, by engaging in conversation outside of their medical condition). The attitudes, mannerisms, and tone of voice of the HCP conveyed compassion. Many participants interpreted clarity of information provided by the HCP as a component of compassion, as it enabled them to have a good understanding about their health condition. Several participants highlighted their preference that HCPs use language that could be easily understood and take the time to explain what is going to happen. One participant reported the following about HCP communication with patients and compassion even when they may not completely understand the experience:

…the doctors talk to you and explain things as if they were an amputee. Like they’re not just saying do this, do that, you know, they kind of – I found it anyway that they really understood it and didn’t make you feel like you were just some, you know, some guy with their leg cut off type thing. Like they understood, kind of understood what I was going through. And did their best to help me in that respect.

[Participant 5]

Participants indicated that not feeling rushed was an important part of making a connection with the HCP. In a virtual appointment, the conversation remained focused on the purpose of the appointment, and the duration of the appointment did not typically exceed what was scheduled. Compared to in-person care, where discussion could veer in many directions and many questions could be asked, some participants felt rushed during virtual appointments. One person shared their views on how feeling rushed may impact the experience of compassion:

That’s just some of the things that was kind of like it just feels like the doctor doesn’t really care or, you know, he’s not compassionate about you. He’s just more compassionate about getting his job done for the day type of thing.

[Participant 4]

### Theme 2: Threats to Compassionate Care in Virtual Rehabilitation: Making a Connection

This theme illustrates the unique challenges of delivering compassionate care in a digital setting based on participants’ experiences with virtual care. From the perspective of some participants, compassionate care was threatened when there was an inability to see the HCP in a virtual appointment. When individuals struggled with technology and required a phone consultation rather than video, some participants suggested this impacted their ability to connect with the provider, as they could not see visual information such as the HCP’s face, facial expressions, and mannerisms. One participant stated:

Well, I think that’s more difficult because it’s just – it’s not on the same level. You know. When you’re standing next to a person or you’re standing next to you, you do have that personal observation, that personal connection and on a screen you don’t.

[Participant 15]

The lack of body language and expressions could make the visit feel impersonal. As such, one participant recommended the importance of an in-person meeting prior to virtual meetings so they could become familiar with the HCP.

Well, I think it would be important that there is a physical meeting before. So, you go in and you visit the physician face-to-face. So, when somebody is talking to you on the phone, it’s not a disembodied voice, you can actually have a picture in your head, what this person looks like, some of their mannerisms so you start to feel more comfortable of what is going to happen. I think if you were to do a virtual – it gets very clinical. It gets very – well, I shouldn’t use the word clinical. But it gets to be very sterile. Here is the question. Answer it. Move on. Next. You know, conclusion, we’re out of here. See you.

[Participant 1]

As a result, most participants strongly preferred video over phone connections, stating the visual information made them feel more comfortable, and several participants linked this to the experience of compassion in virtual care.

Well, you know the whole compassionate aspect, it’s only words really. You can’t see the person. Well, I guess it would be a visual thing. I mean you can’t really see their reaction or, you know. Talking to someone on the phone, you can’t tell if they’re, you know, what their feelings are or whatever.

[Participant 7]

Other participants identified the lack of touch to be a limit to connection in virtual care. This not only limited the HCP’s ability to assess the patient fully but also could limit the connection for some participants as described by one participant:

Like just listen. Listen to their -- listen to their pain. Be sad if they’re sad. Be happy if they’re happy. I’m -- like, you know, I don’t know. I mean you can’t hold them. Like you can’t reach out and touch them. And touch is being the greatest compassion at all that there is. And so, you can’t do that. So, you got to try to do it with your eyes, with your expressions, with your facial expressions, etc.

[Participant 9]

One participant’s account showcased the challenge of delivering compassionate care in a digital setting. The participant shared:

Well, all of the video calls that I had with my doctors, the only one doctor I didn’t like how he handled his was that he used to go by a window frame. As soon as that window’s over, your time’s up type of thing. So, if he gives you a fifteen minute video call or a call or virtual call or whatever you want to call it, and then in that fifteen minutes your time’s up and you didn’t get to say what you wanted to say or the help that you needed wasn’t provided at the time, that’s the only downfall I see in the system…That’s just some of the things that was kind of like it just feels like the doctor doesn’t really care or, you know, he’s not compassionate about you. He’s just more compassionate about getting his job done for the day type of thing…So, that’s the only downfall I had with my virtual appointments.

[Participant 4]

To mitigate some of the innate challenges to providing compassion in virtual care, some participants suggested that prior to delivering virtual care, HCPs should receive training on communication skills about etiquette such as making eye contact, using an interesting background, and not taking calls in non–health care settings. Some participants also discussed the benefits of forming a personal connection to establish rapport with the HCP prior to starting virtual care, for example, via a one-on-one call.

### Theme 3: Facilitating Compassion in Virtual Rehabilitation Through Preparation

In this theme, participants highlighted the impact of adequate preparation by the HCP in advance of the appointment on their experience of compassion. Participant descriptions about preparation for the appointment revolved around 2 aspects of the appointment: the behaviors and actions of the HCP and the logistics surrounding the appointment.

#### Behaviors and Actions of the HCP

Many participants indicated that when HCPs exhibited confidence in the technical skills required to run a virtual appointment or familiarized themselves with patient files and patient history, these actions heightened participants’ feelings of compassion in virtual rehabilitation. One participant illustrated how this was linked to compassionate care:

…But the doctor, you know, he remembered specifically what had happened at last year’s visit…And he remembered the condition of the stump and he specifically asked, you know, did that, you know, has that stayed in that condition and yeah. He just – I think he cares I suppose. I think that’s the overwhelming impression that I get left with… and if he doesn’t remember, he’s at least taken two minutes before getting on the horn and he’s looked through my file and he’s realized what he’s done before, and he can make a comment on it. He familiarized himself with me that’s all. And so, and that, you know, I mean whether or not that’s his actual memory or not, it doesn’t matter. It comes across to me as, you know, as make me feel cared for and important and all that.

[Participant 2]

The experience was also described as more positive and pleasant when the HCP used a visually welcoming background during video calls.

#### HCP and Appointment Logistics

Participants indicated they perceived greater compassion in virtual care when they were clear in advance of their appointment on what to expect during their appointment. Participants felt better prepared when the purpose of the call was clarified, when they were informed about what would be happening during the appointment (eg, whether it will be telephone or video-based, which questions they might be asked, and which exercises they would be doing), and when they received instructions about how to join the call. When participants felt better prepared, they were more relaxed and confident about virtual care.

Some participants described the benefits of offering training to HCPs on the fundamentals of delivering virtual care, noting that such training could streamline HCP behaviors and appointment logistics. For example, one participant explained:

I mean take a Zoom 101 basics course or something like that (laughs) All those goofy mistakes that, you know, there’s not a bright light behind him. All that sort of, you know, practical stuff. I think if you take all the technical stuff out of it and make it easy in that respect, you’re just going to get better results trying to convey any kind of emotion through video.

[Participant 2]

### Theme 4: Benefits of Virtual Care: Fitting Health Care Into Life

This theme describes how delivery of care virtually ensured that participants could fit their health care into their lives, enabling patient autonomy and control. The use of technology enabled participants to receive care while staying home, enabled them to be efficient with their time, and in some instances, offered additional autonomy and independence in their lives. Patients reported shorter wait times for virtual appointments; patients were seen sooner and had a greater number of touchpoints with the HCP, as it was much easier and faster to get to an appointment. Virtual appointments occurred on time as compared to in-person clinic visits, which often ran late. Through virtual care, patients avoided spending time on unnecessary tasks such as driving for long hours; finding parking; using a wheelchair, walker, or other assistive devices; and having someone else drive and attend the appointment, all of which were not required for virtual care. This was convenient for participants, and notably, it granted participants more autonomy and control in their lives since the call could be done independently and did not necessitate, for example, someone to drive them to the appointment. Participants shared the following views about attending appointments virtually:

And honestly, I think that that system is working much better, and it should be better for like I’m pretty sure any patient that’s disabled. Like it would make your life so much easier and it’s like you’re in control. You don’t have to go out of your house, get in a wheelchair or any assisted devices, get into a vehicle, have someone drive you or drive yourself, you know, it’s more of a hassle, right, that you have to leave earlier. So, this is like more convenient for us I guess or not just us, but like for anybody, it’s more convenient that you can do a virtual doctor’s appointment and get the treatment that you need.

[Participant 4]

To me, that was great. I don’t know about other people, but for me, it was great because I didn’t have to try and get ready, try and get dressed. Put on the oxygen. Try and get in the car. Take the walker and trying, try and find where it is in the hospital. And then it’s only like a half hour appointment, then you got to do all the things back home again. So, it was ten times easier for me.

[Participant 9]

While the option of virtual care often promoted autonomy, there were examples of participants still requiring assistance to partake in virtual care. A few participants noted they had the technology available but lacked the knowledge and confidence to use the technology to facilitate the call, especially to initiate the call. As such, they enlisted help from others, including family members and care staff, to facilitate the call or obtain the technology. One participant described:

Well, (laughs), if it wasn’t for my grandchildren and my daughter, I wouldn’t be able to do anything with technology because I’m one of them old farts that just – I can talk on the phone…

[Participant 5]

One participant suggested an approach by which the HCP and their team could assist patients with becoming oriented prior to the scheduled call, enable autonomy, and ensure a successful consultation:

So, it’s just like there should be like an educational assistance, tutorial or a video of what apps or doctors if do use like virtual calls, have the person sit fifteen minutes earlier so they know how to flip the video over on the camera. So, there’s a front camera and a back camera, right. So, like little things like that. I think that – if there was like a little tutorial on that just before your video call it would be fresh in your mind and I guess that’ll help a lot of patients.

[Participant 4]

In most situations, virtual care enabled participants to fit their health care into their daily lives, allowing them to adjust to various circumstances (eg, patients’ roles and responsibilities and weather conditions). For example, one participant highlighted that virtual appointments enabled them to sleep in, take calls in their pajamas, and even be home to receive packages and do other tasks. One participant reflected on the additional conveniences available to him; notably, attending virtually allowed him to continue being available to help his wife, who was also an amputee and to whom he was a caregiver.

In addition, the option of virtual care often enabled patient preferences to be incorporated into their health care. For example, HCPs often accommodated to the patient’s schedule. Some participants noted scheduling an appointment seemed flexible and was not a hassle. One participant reflected on the flexibility:

And they’re always very convenient. That’s the nice thing about the doctors are very flexible with their times. If I’m not available, I found that they’re available at a different time. I think their time is a little bit different schedule for sure. So that is, that is an advantage. That okay, if you can’t talk between 10:00 and 10:15, can we talk to you between 1:00 and 1:15 and that kind of thing. So that’s nice because that makes it feel like it’s not all on you to have everything figure out according to their schedule. It’s still that your personal situation still matters.

[Participant 3]

The option of virtual care also provided safety and comfort to some participants by offering relief from concerns about the COVID-19 pandemic. A few participants described their concerns about being exposed to COVID-19 while attending appointments in person and while waiting for appointments. Participants expressed that virtual appointments offered a sense of relief and comfort and were a safer alternative that allowed them avoid going in to hospitals during the pandemic. For example, one participant noted:

…with the virtual visits I, of course, and being scared of Covid like everybody was, it puts your mind at ease that I didn’t have to take a chance of going down and because it was actually a visit to the hospital.

[Participant 5]

## Discussion

### Principal Findings

This study provides an understanding of the perceptions and experiences of compassion in virtual rehabilitation from the perspective of individuals with ongoing functional disabilities as exemplified by LLA or COPD. These findings extend our understanding of the meaning of compassion from a patient’s perspective and shed light on key elements of compassionate care. By highlighting how compassion can be enacted in clinical encounters and the unique facilitators and threats to compassionate care in digital environments, these findings can be used to inform strategies to support HCPs to optimize the delivery of compassionate virtual care for people in rehabilitation settings.

First, our research provided insight on how participants perceived compassionate care. Our study reported that a feeling of connectedness with the HCP, an HCP’s genuine concern and willingness to help, and patient-centered communication by the HCP are paramount for compassionate care. This is consistent with prior research in which previous definitions of compassion in both face-to-face and virtual health care settings focused on connection and concern for patients. Baguley et al [21] highlighted the importance of connection in a health care setting. They reported that 71% of participants in their sample felt compassion when the practitioner displayed listening and paid attention. A recent qualitative study by Wu and colleagues [22] examined patient-provider communication and compassion in a virtual setting and highlighted the important role of the patient-provider connection. The authors noted that feelings of autonomy and being prioritized were foundational for experiencing compassionate care in a virtual care setting.

Participants identified unique threats to compassion in a digital environment, including lack of connection with the HCP. We also identified new insights about the ways in which HCP actions navigating virtual care during appointments; appointment logistics and HCP preparedness for consultations enhance patient comfort and feelings of compassion during a virtual visit. Study findings are consistent with recent work by Desveaux and colleagues [23] in a primary care setting, which illustrates that a provider’s behaviors are linked to how compassionate care is perceived by patients.

Wu and colleagues [22] suggest the importance of virtual training in offering clinicians the requisite technical, examination, and communication skills for positively impacting their comfort and preparedness for virtual interactions with patients. Such strategies may help to address some of the threats to compassionate care imposed by virtual care by enabling people to have successful virtual visits. Delivering compassionate care may call for structured preparation and training of HCPs to deliver virtual rehabilitation (eg, digital skills and communication skills in a digital environment). Future work is needed to explore best practices to facilitate compassionate behaviors in virtual care and the optimal models for training HCPs for compassionate virtual care.

Interestingly, participant perspectives reported in our study suggest an interplay between compassion and autonomy and comfort experienced in virtual rehabilitation. Participants identified several benefits of virtual care, including that wait times were shorter, they expended less time and energy to attend virtual appointments as compared to in-person care, and appointment times were more flexible. This is consistent with literature highlighting the convenience of virtual care [5,6]. However, our findings suggest that the benefits go beyond convenience for populations with complex health needs by enhancing a sense of control, autonomy, and independence, with less disruption to other aspects of daily life.

### Study Limitations

For pragmatic reasons, we recruited a convenience sample of individuals who attended a virtual appointment. This method may not have captured those who faced major barriers and were unable to access virtual care. There was variation in the mode of virtual care and types of virtual appointments that participants had attended (eg, phone and video) and the nature of the activities that were being completed during the virtual appointment (eg, physiotherapy and consultation with a nurse or doctor). While participants received at least one virtual rehabilitation consultation, some participants had additional virtual interactions, for example, as a result of participating in virtual exercise in a group setting. We did not ask study participants about the total number of virtual appointments they attended, and thus, could not consider how their level of experience and comfort with tools and technology may impact their perspective about their virtual care interaction. Findings may not be transferable to other settings or populations due to the unique needs and experiences of the participants in this study. Moreover, participants who opted for virtual care may have different characteristics than those individuals who did not or could not access virtual care.

### Conclusion

Our study identified key aspects of the patients’ perception of compassionate care in virtual rehabilitation settings. Ensuring that HCPs are prepared and adequately trained in forming personal connections with patients, communicating effectively in a virtual setting, and using the tools necessary to facilitate the connection prior to initiating a patient-provider relationship in a virtual setting will contribute to this being a positive patient experience. Future work could explore whether virtual care should be introduced only when extending an existing face-to-face patient-provider relationship and whether virtual care might be reserved to address only certain needs within the care journey. Potential changes to patients’ attitudes and perceptions about virtual care as they progress through their care journey and develop rapport with their HCP could also be explored.

## Abbreviations

COPD: chronic obstructive pulmonary disease
COREQ: Consolidated Criteria for Reporting Qualitative Research
HCP: health care provider
LLA: lower limb amputation
